# Nuclear βII-Tubulin and its Possible Utility in Cancer Diagnosis, Prognosis and Treatment

**DOI:** 10.3389/fcell.2022.870088

**Published:** 2022-05-30

**Authors:** Richard F. Ludueña, Consuelo Walss-Bass, Anna Portyanko, Jiayan Guo, I-Tien Yeh

**Affiliations:** ^1^ Department of Biochemistry and Structural Biology, University of Texas Health Science Center at San Antonio, San Antonio, TX, United States; ^2^ Department of Psychiatry and Behavioral Sciences, University of Texas Health Science Center at Houston, Houston, TX, United States; ^3^ Belarussian State Medical University, Minsk, Belarus; ^4^ Fosun Pharma, Shanghai, China; ^5^ Department of Pathology, University of Texas Health Science Center at San Antonio, San Antonio, TX, United States

**Keywords:** Beta-II-Tubulin, nucleus, cancer diagnosis, Cancer prognosis, cancer treatment, CRISPR-Cas9

## Abstract

Microtubules are organelles that usually occur only in the cytosol. Walss et al. (1999) discovered the βII isotype of tubulin, complexed with *α*, in the nuclei of certain cultured cells, in non-microtubule form. When fluorescently labeled tubulins were microinjected into the cells, only *αβ*II appeared in the nucleus, and only after one cycle of nuclear disassembly and reassembly. It appeared as if *αβ*II does not cross the nuclear envelope but is trapped in the nucleus by the re-forming nuclear envelope in whose reassembly *β*II may be involved. *β*II is present in the cytoplasm and nuclei of many tumor cells. With some exceptions, normal tissues that expressed βII rarely had βII in their nuclei. It is possible that βII is involved in nuclear reassembly and then disappears from the nucleus. Ruksha et al. (2019) observed that patients whose colon cancer cells in the invasive front showed no βII had a median survival of about 5.5 years, which was more than halved if they had cytosolic *β*II and further lessened if they had nuclear *β*II, suggesting that the presence and location of *β*II in biopsies could be a useful prognostic indicator and also that *β*II may be involved in cancer progression. Yeh and Ludueña. (2004) observed that many tumors were surrounded by non-cancerous cells exhibiting cytosolic and nuclear *β*II, suggesting a signaling pathway that causes *β*II to be synthesized in nearby cells and localized to their nuclei. *β*II could be useful in cancer diagnosis, since the presence of *β*II in non-cancerous cells could indicate a nearby tumor. Investigation of this pathway might reveal novel targets for chemotherapy. Another possibility would be to combine *αβ*II with CRISPR-Cas9. This complex would likely enter the nucleus of a cancer cell and, if guided to the appropriate gene, might destroy the cancer cell or make it less aggressive; possible targets will be discussed here. The possibilities raised here about the utility of *β*II in cancer diagnosis, prognosis, biology and therapy may repay further investigation.

## Introduction

Microtubules are composed of the protein tubulin, which is a heterodimer of two subunits, designated *a* and *ß* (Ludueña et al., 1977). Both *a* and *ß* consist of multiple isotypes, each of which is encoded by different genes; the amino acid sequence differences among the vertebrate isotypes have been highly conserved in evolution, suggesting that they may be functionally significant (Ludueña, 2013). In this study we have focused on the *ß* isotypes. These isotypes exhibit major differences in their tissue distributions. Under normal circumstances, the βI and βIV isotypes are widely distributed among cells and tissues (Roach et al., 1998). In contrast, βII is highly expressed in nerves, both in neurons and supporting cells such as glia, and also in muscle cells, while βIII is particularly common in neurons (not in glia) and also in the testes (Burgoyne et al., 1988; Lewis and Cowan, 1988). The *β*V isotype is widespread but not predominant, being slightly higher in a few cell types (Nakamura et al., 2004; Chao et al., 2012). The *β*VI isotype is restricted to hematopoietic tissues, such as bone marrow, platelets and non-mammalian erythrocytes (Murphy and Wallis, 1983; Wang et al., 1986). Although every *ß* isotype can participate in some of the main microtubule functions, such as forming the mitotic spindle and mediating intracellular transport (Lopata and Cleveland, 1987), our results and those of others have indicated that there is some specialization. *β*II appears to be associated with membrane rearrangements, such as neurite outgrowth (Guo et al., 2010; Guo et al., 2011), while *β*III, when mixed with small amounts of other *ß* isotypes, forms very dynamic microtubules (Panda et al., 1994; Vemu et al., 2016) and also protects cells from various stresses (Gan et al., 2007; Guo et al., 2010; Guo et al., 2018). In addition, studies on purified *αβ*II, αβIII and αβIV dimers have shown that the anti-tumor drugs vinblastine and taxol interact most strongly with *αβ*II and most weakly with *αβ*III (Derry et al., 1997; Khan and Ludueña, 2003).

In cancer cells, the expression of the *ß* isotypes changes dramatically. Many cancer cells express *β*II and the most aggressive cancers express *β*III, regardless of the extent to which either of these is expressed in the non-transformed cells from which these cancers originated (Katsetos et al., 2003; Yeh and Ludueña, 2004).

## Nuclear *β*II

**FIGURE 1:**
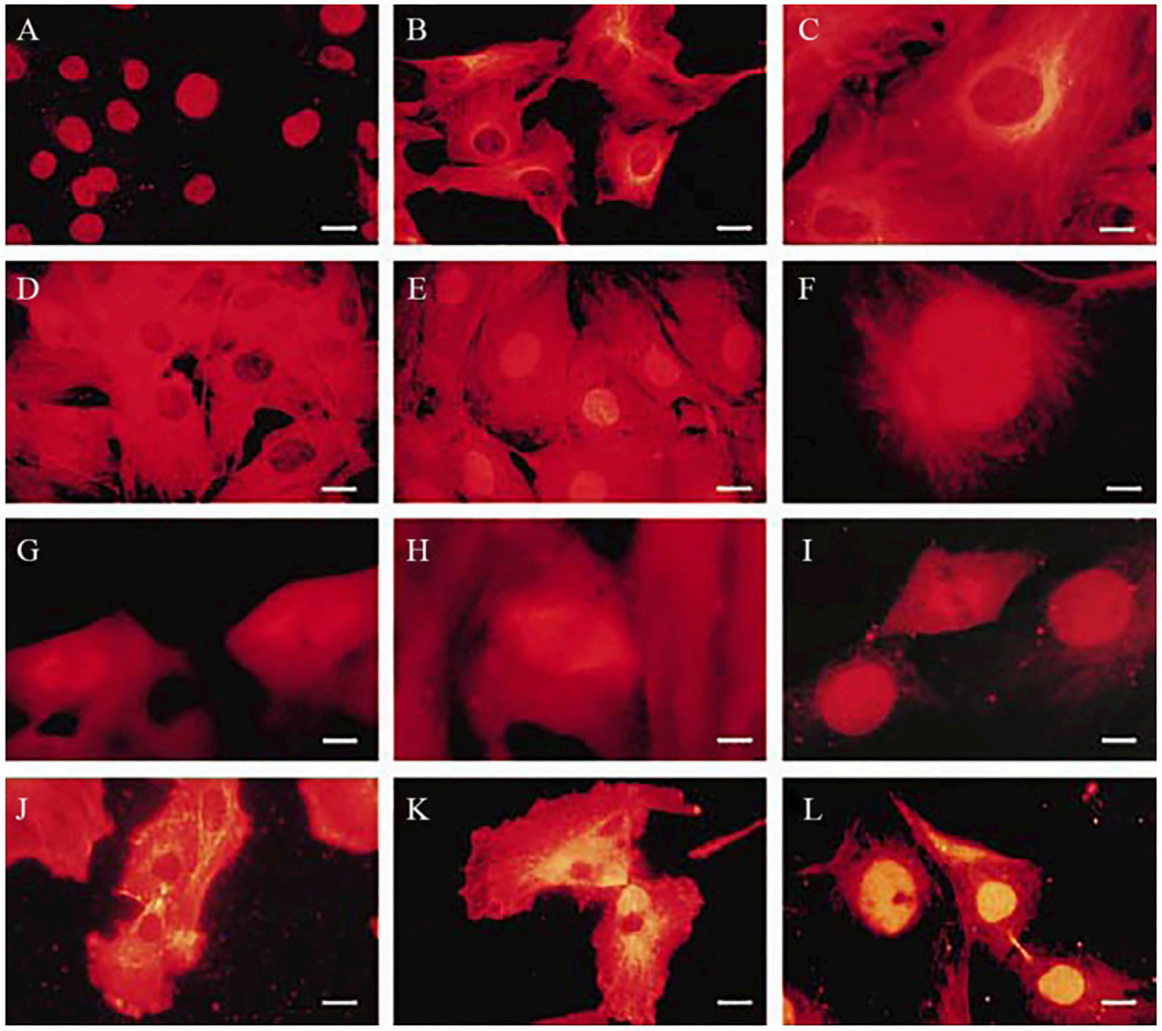
Detection of *ß*-tubulin isotypes during the cell cycle of cultured rat kidney mesangial cells by indirect immunofluorescence. **(A)** Interphase cells treated with anti- *ß*II (0.03 mg/ml). Note that only the nuclei are stained. **(B)** Interphase cells treated with anti- *ß*I (0.1 mg/ml). Note very little staining in the nuclei. **(C)** Interphase cells treated with anti-βIV (0.17 mg/ml). Note very little staining in the nuclei. **(D)** Cells treated with 30 μg/ml nocodazole, stained with anti-βI (0.1 mg/ml). Note widespread staining except in the nuclei. **(E)** Cells treated with 30 μg/ml nocodazole, stained with anti-*ß*II (0.1 mg/ml). Note staining in the nuclei. **(F)** Cell during prophase, treated with anti-*ß*II (0.05 mg/ml). Note that asters are beginning to form and that they contain *ß*II. **(G)** Cells during metaphase, treated with anti-*ß*I (0.1 mg/ml). *ß*I is in the spindle. **(H)** Cell during metaphase, treated with anti-*ß*IV (0.17 mg/ml). *ß*IV is in the spindle. **(I)** Cell during metaphase, treated with anti-*ß*II (0.05 mg/ml). *ß*II is in the spindle. **(J)** Cell during late telophase, treated with anti-*ß*I (0.05 mg/ml). *ß*I is not in the re-forming nuclei. **(K)** Cell during late telophase, treated with anti-*ß*IV (0.08 mg/ml). *ß*IV is not in the re-forming nuclei. **(L)** Cell during late telophase, treated with anti-*ß*II (0.05 mg/ml). Note that the nuclei, staining brightly for *ß*II, have now re-formed. Bar = 28 μm (From. Walss et al., 1999).

The nuclear *β*II isotype was first observed in rat kidney mesangial cells (Walss et al., 1999). Nuclear βII was bound to *a* and was normal in that it bound to drugs such as taxol, vinblastine and colchicine (Walss et al., 1999; Xu and Ludueña, 2002; Walss-Bass et al., 2003). Nuclear *β*II was not in the form of microtubules but rather appeared to be in small bodies, whose internal organization was unclear (Walss et al., 1999). During mitosis, *β*II participated in forming the mitotic spindle and then was back in the nucleus by interphase (Walss et al., 1999) (Figure 1). Figure 1 also shows that, although *β*I and *β*IV are also present in these cells, only *β*II ends up in the nucleus. Microinjection of fluorescently labeled *αβ*II, *αβ*III, and *αβ*IV indicated that only *αβ*II entered the nucleus and only after one cycle of nuclear assembly and disassembly (Walss-Bass et al., 2001), suggesting that, rather than penetrating the nuclear envelope, *αβ*II was in the nucleus while the envelope re-formed around it, consistent with the finding that *β*II-tubulin appears to have a specific connection to heterochromatin protein 1 and to the nuclear envelope (Kourmouli et al., 2001). In general, microtubules are restricted to the cytosol, so it is unusual to see apparently viable tubulin in the nucleoplasm.

## Nuclear βII in Cancer Prognosis

**FIGURE 2:**
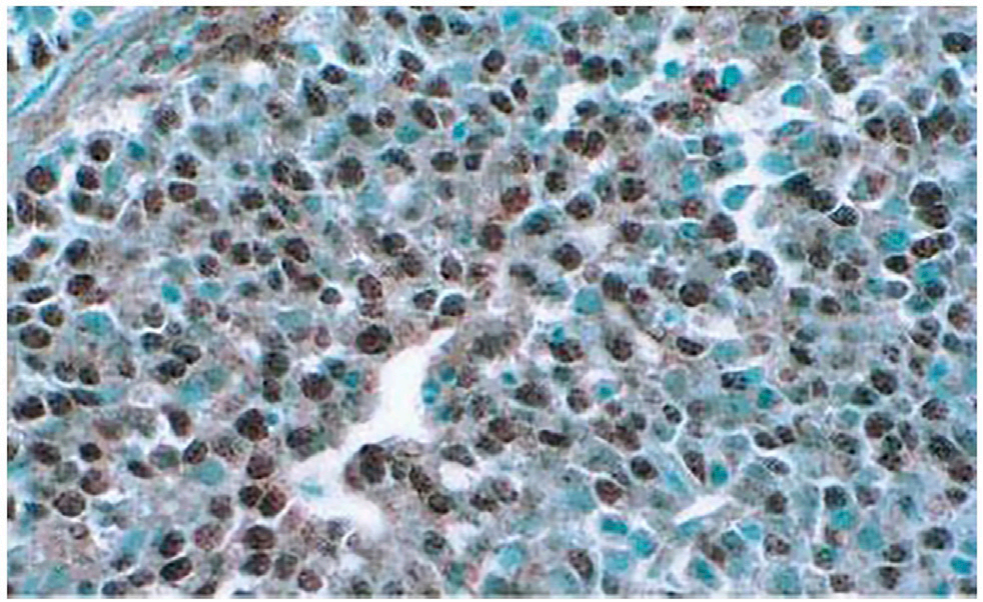
Carcinoma of the Ovary stained for *ß*II. A monoclonal antibody to *ß*II was the primary antibody followed by a rabbit anti-mouse antibody and then Streptavidin horseradish peroxidase, followed by diaminobenzidine and OsO_2_. Brown color indicates the location of *β*II. Note that most of the cell nuclei stain for *β*II. Methyl green, which binds to DNA, was used as the counter-stain. Note that a few nuclei do not have *β*II (From Yeh and Ludueña, 2004).

**FIGURE 3:**
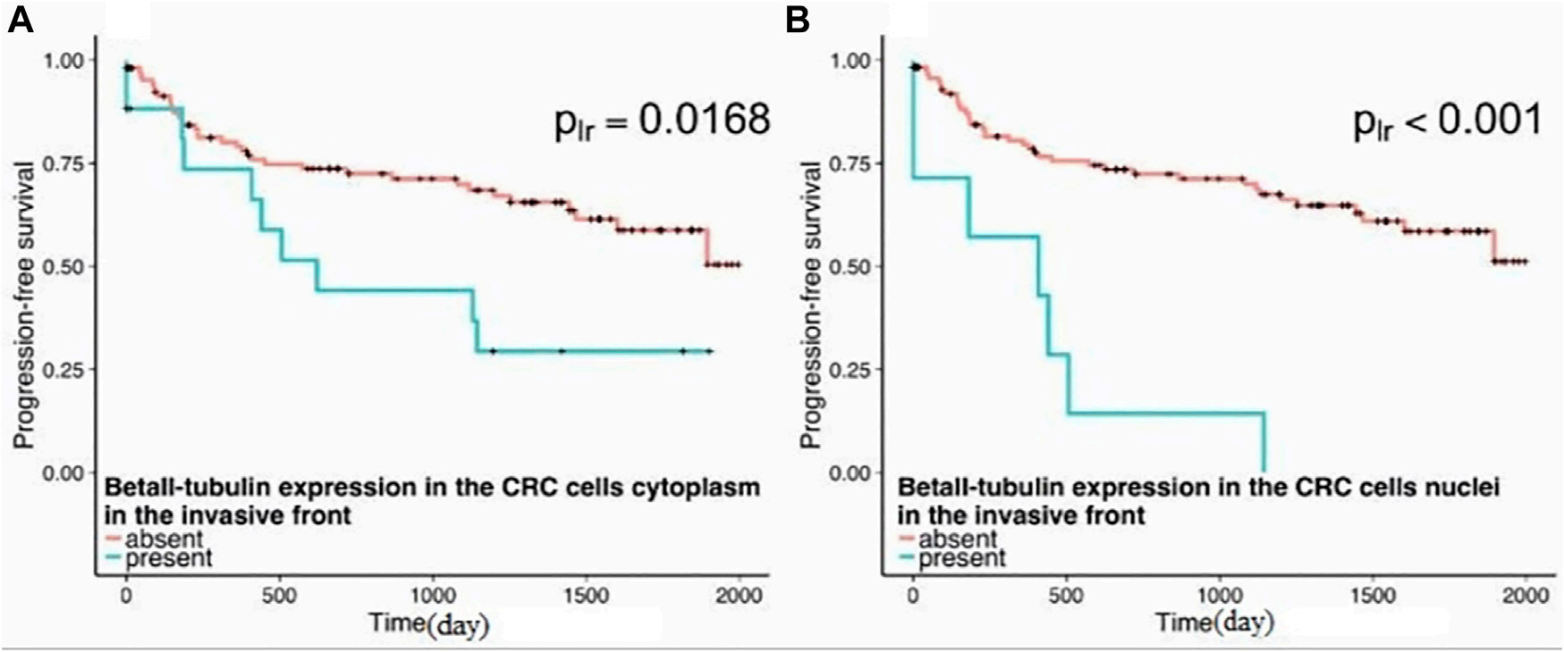
Progression-free survival in patients as a function of the presence of *β*II-positive staining in the invasive front of colorectal cancer tumors. **(A)** The progression-free survival is decreased in patients with the presence of cytoplasmic *β*II-tubulin in the invasive front (plr = 0.0168). **(B)** Patients with the presence of nuclear βII tubulin staining in the invasive front demonstrate worse prognosis in comparison with patients without positive staining in the nuclei (plr< 0.001) (From Ruksha et al., 2019).

A study of a large number of cancers from multiple patients showed that many of them expressed *β*II in the cytosol and most also had *β*II in the nuclei (Yeh and Ludueña, 2004; Ruksha et al., 2019) (Figure 2). This may partially explain why drugs such as vinblastine and taxol, which interact more strongly with *β*II (Derry et al., 1997; Khan and Ludueña, 2003), are useful agents in cancer chemotherapy, and also why they cause substantial neuropathy (Markman, 2003; Mukhtar et al., 2014). A study of colon cancer patients (Ruksha et al., 2019) indicated that patients whose cancer cells in the invasive front showed no *β*II had a median survival rate of about 5.5 years, while those whose cells had only cytosolic *β*II had a median survival of just under 2 years. Those with nuclear *β*II had a 5-year survival rate of zero and a medial survival of 13 months (Figure 3). It would appear that the presence of *β*II and its localization in the nuclei, as seen in a tumor biopsy or in an excised tumor, would be useful prognostic indicators of the survival of the patient. These results also raise the possibility that *β*II expression in the nuclei may be necessary for cancer progression. On the other hand, Yeh and Ludueña (2004) also found that some normal tissues also contained cells with nuclear *β*II, although the frequency of occurrence was generally fairly low. This raises the possibility that nuclear *β*II may play a role in re-formation of the nuclear envelope after mitosis, even in normal cells, after which it disappears; this would be consistent with the observations of,Kourmouli et al. (2001) about *β*II being associated with proteins that bind to the nuclear envelope, although their results do not rule out the possibility of the *β*I and *β*IV isotypes having equally tight binding to the nuclear envelope.

## Nuclear βII in Cancer Diagnosis

**FIGURE 4:**
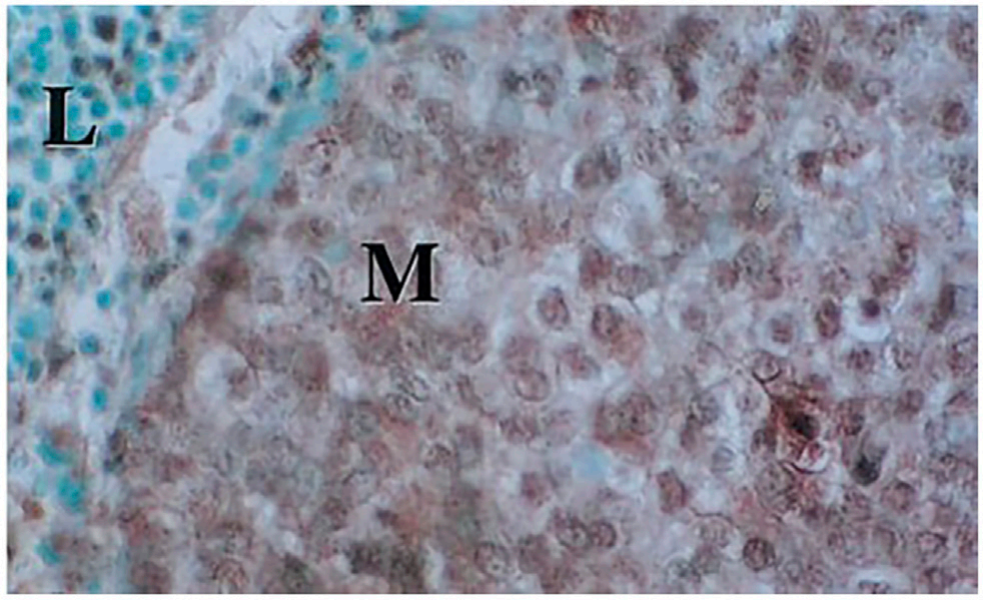
Lymph node with metastatic breast carcinoma. Sample was processed as in Figure 2. Note both cytoplasmic and nuclear staining for βII in the metastatic tumor cells (M) and some weak nuclear staining for βII in the adjacent lymphocytes (L). (From Yeh and Ludueña, 2004).

We observed that many tumors were surrounded by zones of non-transformed cells that contained substantial amounts of cytosolic *β*II and also of nuclear *β*II (Yeh and Ludueña, 2004; Ruksha et al., 2019) (Figure 4). These often included lymphocytes as well (Yeh and Ludueña, 2004). These results raise the possibility that, by some as yet unknown mechanism, tumor cells cause nearby cells to express *β*II-tubulin and localize it to their nuclei. This also suggests a potential role in cancer diagnosis. If a biopsy probe misses the tumor itself, resulting in an apparent false negative, then it might pull out cells close to the tumor that contain cytosolic and perhaps nuclear *β*II. This would not only enhance the power of the biopsy, but perhaps even give an indication of the grade of the tumor. The outcome would be the possibility of earlier diagnosis of the tumor and an increased chance of treating the patient successfully.

## Nuclear βII in Cancer Therapy

The results described above strongly suggest that expression of *β*II in cancer cells and its localization in their nuclei serve a function required by the cancer cell. This function would probably involve some kind of signaling pathway. A microarray experiment may be able to identify the components of this pathway. Drugs could then be developed to inhibit a key pathway component and thus slow or block the progression of the cancer.

## Could Nuclear βII be Combined With CRISPR-Cas9 to Create a Novel Anti-Tumor Therapy?

CRISPR-Cas9 is an agent that, when combined with a guideRNA (gRNA), can inactivate targeted genes, as determined by the gRNA; the strategy is to “correct” mutations that cause hereditary diseases (Naso et al., 2017). Some recent attempts to treat cancer involve using CRISPR-Cas9 to “correct” mutations in oncogenes (Li et al., 2020).

The approach we are suggesting here does not involve “correcting” any mutations, but rather targeting specific genes only in the cancer cell. One might imagine that combining CRISPR-Cas9 with *β*II-tubulin could create an agent that could enter the nuclei of cancer cells. For any agent involving CRISPR-Cas9, there would be three questions that need to be answered:

First, how could a *β*II-CRISPR-Cas9 complex enter the target cell? If there is a receptor on the tumor cell membrane then it may be possible to add to the complex a factor that would bind to the receptor, for example, a lipid nanoparticle directed at the liver; this has been used with CRISPR-Cas9 to treat transthyretin amyloidosis (Finn et al., 2018; Gillmore et al., 2021). Otherwise, it may be necessary to encapsulate the complex in something that would bind to many types of cells, such as an adeno-associated virus (Naso et al., 2017). In addition to potential immunological issues, this could pose potential dangers that will be discussed below.

Second, how could a *β*II-CRISPR-Cas9 complex enter the nucleus? It is likely that this would require that the *β*II in the complex be bound to *a*-tubulin, so that the *β*II is in its normal functional conformation. If this is feasible, entry of the complex into the nuclei could be the easiest of the steps. Just as is the case with αβII, the nucleus would re-assemble around it (Walss et al., 1999). No nuclear recognition signal would be required. This is likely to happen in many cancer cells, but not in many normal cells. However, the fact that we found some normal cells with nuclear *β*II (Yeh and Ludueña, 2004) suggests that the *αβ*II-CRISPR-Cas9 complex could end up in the nuclei of some normal cells as well. We do not as yet know the precise function that nuclear *β*II serves in the normal cells where it occurs but it may play a role in reassembly of the nuclear envelope as suggested by Kourmouli et al. (2001) after which it could be degraded. If this is so, this process could occur in some normal tissues, at least in the ones where we have observed nuclear *β*II, such as bone marrow (Yeh and Ludueña, 2004). Cells such as neurons would virtually never reproduce and that may explain why we have not seen nuclear *β*II in these cells. In short, it is possible that some normal cells may contain nuclear *β*II for a very brief time and that the *αβ*II-tubulin-CRISPR-Cas9 complex could enter the nuclei of these cells, and possibly cause some damage.

The issue of targeting the *αβ*II-tubulin-CRISPR-Cas9 complex to enter the nucleus is complicated by the fact that we do not know the precise role of nuclear *β*II and that the results at this point are not consistent with an absolute requirement for nuclear *β*II in cell division. One the one hand, Yeh and Ludueña (2004), looking at excisions of tumors from human patients, found that nuclear *β*II was present in every sample (although not every tumor cell) of tumors of the stomach, colon, bone, and prostate and in at least half of the tumors of the pancreas, lung, lymphocytes, ovary and breast. On the other hand, nuclear *β*II was rarer in melanomas, squamous cell carcinomas, and brain tumors. This is consistent with the observation that mitosis-specific drugs are less effective in cancer chemotherapy than are those that target microtubules, which have numerous functions in cells (Komlodi-Pasztor et al., 2011; Mitchison, 2012). Similarly, normal bone marrow cells, which reproduce more rapidly than some cancer cells (Mitchison, 2012) showed some nuclear βII (Yeh and Ludueña, 2004); however, other normal tissues, such as small bowel and colon, which one would expect to have high rates of reproduction, showed none (Yeh and Ludueña, 2004; Ruksha et al., 2019). In short, if nuclear *β*II plays an important role in reproduction in some cells, one cannot conclude that this role is universal, and if a tubulin is required for this role, it is possible that in certain cells, including some tumor cells, some other tubulin isotype may serve. In other words, it is possible that there are tumors into whose nuclei an *αβ*II-CRISPR-Cas9 complex might not enter. It is possible, however, that a biopsy or other assay might determine if a given tumor contains nuclear βII, in which case the likelihood of the complex entering the nucleus would probably be higher.

Third, what would the gRNA target? There are several possibilities:

A)The gRNA could target the genes for housekeeping proteins, such as aldolase or any component in the glycolytic pathway or Krebs cycle. This would virtually ensure the death of the cell, which would be unable to metabolise. For normal cells, this could be catastrophic, so toxicity could be a serious problem.
B)The gRNA could target genes encoding proteins involved in cell reproduction, such as DNA polymerase. This would prevent the cancer from growing. However, many normal cells need to reproduce and hence this could cause long-term problems such as loss of bone marrow or intestinal epithelia.
C)The gRNA could target the *β*III-tubulin isotype. This could be the most beneficial outcome. First, it would be the least likely to have disadvantageous side effects, since only two normal tissues express substantial amounts of *β*III. Neurons do not have nuclear *β*II and hence the synthesis of *β*III would not be affected. The other tissue is the testis, which could be a problem, but not for women. One of the great advantages of a gRNA targeting *β*III is that it could prevent cancer progression. As described above, many cancers express both *β*II and *β*III. The expression of *β*II makes them susceptible to treatment with anti-tumor drugs such as taxol and vinblastine (Derry et al., 1997; Khan and Ludueña, 2003), but cancers can often escape that by making more *β*III (Mozzetti et al., 2005). If *β*III synthesis is blocked then it is possible that lower doses of taxol and vinblastine, causing less neuropathy, may be sufficient to treat the tumor successfully.

## Potential Problems and Their Potential Solutions

The first potential problem to be faced would be technical: the construction of the *αβ*II-CRISPR-Cas9 complex in such a way that the *αβ*II is linked to the rest of the complex without being denatured. One might begin by making recombinant human *αβ*II tubulin. This has already been accomplished for human *αβ*III tubulin (Vemu et al., 2016), so making recombinant human *αβ*II should not be too difficult. The linking of the dimer to the CRISPR-Cas9 complex could perhaps be accomplished using one of many available chemical cross-linkers. Many of these target sulfhydryl groups, but this could be a problem since the sulfhydryl group of C239 in *ß*-tubulin is very reactive and its reaction inhibits microtubule assembly and probably makes the tubulin molecule non-functional (Palanivelu and Ludueña, 1982; Little and Ludueña, 1985; Bai et al., 1989). Reaction of the sulfhydryl group is inhibited by any drug binding to the colchicine site (Ludueña and Roach, 1991). It would probably be better to use a cross-linker targeting amino groups. One such reagent, dimethyl-3,3’-(tetramethylenedioxy) dipropionimidate dihydrochloride (DTDI), was used to covalently link α to *ß* and the reaction was actually enhanced by either colchicine or vinblastine, suggesting that the DTDI reaction was not blocking access to important sites on the tubulin molecule as well as raising the possibility that it could stabilize the conformation of the tubulin (Ludueña et al., 1977). Perhaps that reagent could also be used to link αβII to the CRISPR-Cas9 complex. Obviously, a good deal of experimentation to optimize the reaction would be required.

The tubulin molecule has long been known to have an unstable conformation (Wilson, 1970); it spontaneously denatures when it is in solution (Schwarz et al., 1998) and this needs to be considered in the methodology proposed here. However, the different isotypes have different stabilities. Using a variety of approaches, it appears safe to say that the conformation of the *αβ*II dimer, although not as stable as that of the *αβ*III dimer, is more stable than that of the *αβ*IV dimer (Banerjee et al., 1994; Sharma and Ludueña, 1994; Banerjee et al., 1997; Schwarz et al., 1998). Also, the observations of Walss et al. (1999) indicate that the *αβ*II dimer can go from its nuclear state, forming a small body, possibly a filament, to forming the microtubules of the mitotic spindle and then returning to whatever state it was in when it reappears in the nucleus; presumably, the conformation of the *αβ*II dimer survives intact through all of these changes, thereby speaking to some degree of stability. That the nuclear *αβ*II dimer is in a normal conformation is supported by the observation that it can bind to colchicine, vinblastine and taxol (Walss et al., 1999; Xu and Luduena, 2002.; Walss-Bass et al., 2003). Second, the *αβ*II dimer has to be able to survive being linked to the CRISPR-Cas9 complex. At the moment, we can safely say that the *αβ*II dimer retains its conformation after being linked to the fluorescent marker 5-(4,6-dichlorotriazin-2-yl)aminofluorescein (DTAF) (Walss et al., 1999). At any stage during this process, the intactness of the tubulin conformation can be assayed by test of its ability to bind to colchicine (Borisy, 1972).

It may be difficult to find a way to ensure that the *αβ*II-CRISPR-Cas9 complex enters the target cell. It would depend on how the complex is packaged. If it is something that would allow the complex to enter any cell type, then there may be too many side effects, even if the cancer is neutralized, although these could perhaps be controllable, as will be described below. Also, it is not possible *a priori* to estimate how many of the cancer target cells would be penetrated by the complex. A narrower approach would be to target the tumor to a cell-type-specific receptor. An example would be the CD20 protein on the surface of B-cell lymphocytes (Pavlasova and Mraz, 2019). If the αβII-CRISPR-Cas9 complex could be targeted to CD20 and then somehow internalized into the B-cell, the mechanism of action of the complex would be more lethal to cancerous B-cells than to normal B-cells and thus, the immune system of the patient is more likely to remain functional. This is analogous to the mechanism of action of CD20-binding monoclonal antibodies, such as rituximab, useful in treating many hematologic tumors (Saini et al., 2011), although its action against the target cell is external rather than internal. Other tumors may be in tissues that express analogous surface antigens and the same logic could apply.

Another problem is that the *αβ*II-CRISPR-Cas9 complex may enter the nuclei of normal cells, some of which clearly have nuclear *β*II (Yeh and Ludueña, 2004), and may thus be toxic. This could perhaps be prevented by having the αβII-CRISPR-Cas9 complex self-destruct. Since the *αβ*II only appears to enter the nucleus during mitosis, and if in a given cell type, the interval between divisions is longer, then it may be advantageous to have the complex disappear. If the *αβ*II-CRISPR-Cas9 complex were modified in some way so that the ubiquitin system could degrade it after an interval of time, the probability of the complex lasting long enough to do serious damage to a slow-dividing cell will be greatly diminished. The modification could be as simple as engineering an arginine onto the N-terminus, since this residue allows for rapid degradation (Varshavsky, 1996). Perhaps even better, since most tubulins begin with a Met-Arg sequence, once could simply remove the N-terminal methionine, leaving the arginine as the new N-terminus. Slower degradation could be mediated by putting in a glutamate instead (Varshavsky, 1996). Similarly, the C-terminal end could be modified in any of a number of ways to favor degradation (Chatr-Aryamontri et al., 2018)). In short, it may be possible to “fine-tune” the rate at which degradation occurs so as to have the *αβ*II-CRISPR-Cas9 complex remain in the cytosol long enough to enter the nucleus of a rapidly dividing tumor cell, but not so long that it might do the same in a normal cell. Since some tumor cells could divide slowly and some normal cells divide quickly, this would have to be carefully addressed. It may be advisable to do the modifications on the *a*-tubulin subunit, since the *β*II subunit should arguably remain intact to be able to remain in the nucleus. This fine-tuning approach could also address another possible drawback, namely that the Cas9 endonuclease component of the *αβ*II-CRISPR-Cas9 complex, even with the appropriate gRNA, may cause nonspecific damage to more than just the target genes, a process called chromothripsis (Leibowitz et al., 2021) and thus could be too toxic.

Of the various potential targets for the gRNA described above, the most intriguing one, as mentioned above, is *β*III, because very few normal cells, except for neurons and the testis, appear to express it, and because it is produced by so many aggressive cancers. However, there are reports of *β*III being expressed in non-neuronal cells at low levels and participating in forming the mitotic spindle (Jouhilahti et al., 2008). Again, just as described above, designing the *αβ*II-CRISPR-Cas9 complex to have a limited lifetime could cope with this issue as well. Finally, if the complex only lowers *β*III expression levels rather than eliminating *β*III altogether, treatment could consist of the complex combined with a drug favoring binding to *β*III. Several such drugs have been designed to bind to the colchicine site on *β*III (Pallante et al., 2020); one of these is more effective than paclitaxel on a *β*III-overexpressing human breast cancer cell line in a transgenic mouse model (Yeh et al., 2016). Similarly, the taxane cabazitaxel is more effective than docetaxel at inhibiting dynamics *in vitro* of microtubules that contain *β*III-tubulin than of microtubules that lack *β*III (Smiyun et al., 2017) and appears to be a useful treatment in advanced prostate cancer (Wallis et al., 2021). These or similar drugs might be therapeutically useful in combination with the *αβ*II-CRISPR-Cas9 complex proposed here.

A potentially more serious problem, however, arises from the *β*V isotype, which is fairly closely related to *β*III. Although the αβV dimer has never been purified and its specific properties determined, the fact that *β*V shares two unusual features with *β*III: a cysteine cluster (C124, C127, C129), and the lack of the easily oxidized C239 (Bai et al., 1989), together with its ability to make microtubules disassemble rapidly *in vivo* (Bhattacharya and Cabral, 2004, 2009) raise the possibility that *β*V may be able to protect cells from oxidative stress and also form dynamic microtubules. In short, *β*V may easily substitute for *β*III in a cancer cell and keep the cell viable despite treatment with an *αβ*II-CRISPR-Cas9 complex with a gRNA directed against *β*III. In other words, it is possible that silencing of *β*III by the complex could result in over-expression of *β*V by the cancer cell and the cancer would continue to grow and spread; in fact, some observations suggest that cancers can over-express either *β*III or *β*V (Hiser et al., 2006; Cucchiarelli et al., 2008; Leandro-García et al., 2010; Chao et al., 2012). One mitigating circumstance is that over-expression of *β*V does not seem to be so strongly associated with aggressiveness as is the case with *β*III (Christoph et al., 2012). On the other hand, over-expression of *β*V may be associated with increased resistance to taxanes (Işeri et al., 2010). A possible solution to this would be to have a second *αβ*II-CRISPR-Cas9 complex with a gRNA directed against *β*V. Alternatively, if more were understood about the drug-binding properties of *β*V, perhaps a *β*V-specific drug could be designed as a backup chemotherapeutic agent.

## Summary and Prospectus

The data presented here suggests very strongly that nuclear *β*II expression in and near cancer cells could be useful in the diagnosis and prognosis of cancer. It is also potentially possible that elucidating the pathway leading to over-expression of *β*II in cancer cells and its localization to the nuclei could lead to development of novel cancer treatments. It cannot be emphasized enough, however, that the concept of using an *αβ*II-CRISPR-Cas9 complex to treat cancer is entirely hypothetical. In addition to the potential problems outlined above, it is possible that an *αβ*II-CRISPR-Cas9 complex may be too large and unwieldy to enter a cancer cell, let alone do the job we hope for in the nucleus of that cell. However, it could not hurt to try and, if the arguments made above are borne out, such a complex could become a novel and useful treatment for cancer.

## Data Availability

The original contributions presented in the study are included in the article/Supplementary Material, further inquiries can be directed to the corresponding author.
